# Exercise and neural circuit stability in early Alzheimer’s disease: evidence across memory, executive, cholinergic, and circadian systems

**DOI:** 10.3389/fnagi.2026.1863929

**Published:** 2026-06-23

**Authors:** Qing Li, Mingchen Gu, Wei Li

**Affiliations:** Jiangsu College of Nursing, Huai’an, Jiangsu, China

**Keywords:** Alzheimer’s disease, EC–DG circuit, exercise, MS/VDB–hippocampal circuit, neural circuits, SCN–PVN axis, synaptic plasticity, vHPC–mPFC pathway

## Abstract

Early Alzheimer’s disease (AD) is increasingly understood as a phase in which neural circuit dysfunction emerges alongside molecular pathology and local neuronal injury. Although physical exercise has been linked to a deceleration in cognitive decline and beneficial cognitive and neural outcomes, most mechanistic explanations have predominantly focused on molecular or cellular pathways, with less emphasis on circuit-level interpretations. This narrative review integrates evidence from four neural systems that exhibit early vulnerability in AD and are pertinent to exercise-responsive cognitive or behavioral domains: the entorhinal cortex–dentate gyrus (EC–DG) circuit, the ventral hippocampus–medial prefrontal cortex (vHPC–mPFC) pathway, the medial septum/vertical diagonal band–hippocampal (MS/VDB–hippocampal) cholinergic circuit, and the suprachiasmatic nucleus–paraventricular nucleus (SCN–PVN) circadian axis. Across these systems, the evidence remains inconsistent. Direct circuit-level findings, including electrophysiological and functional-connectivity measurements, indicate circuit disruption in specific domains, whereas many exercise-related effects are inferred from molecular, structural, neurochemical, behavioral, or clinical-proxy outcomes. We therefore propose a multicircuit framework in which exercise may ameliorate AD-related cognitive and behavioral dysfunction through convergent but circuit-specific routes: EC–DG excitability and plasticity, vHPC–mPFC communication substrates, MS/VDB–hippocampal cholinergic modulation, and SCN–PVN circadian-neuroendocrine timing. This framework clarifies the evidence boundaries of exercise-related circuit modulation in early AD and identifies direct circuit-level measurements as a priority for future translational studies.

## 1 Introduction

AD is the most common neurodegenerative cause of dementia and remains a major public health challenge worldwide (Livingston et al., 2020). Clinically, AD is characterized by progressive impairments in memory, executive function, and behavior that ultimately compromise independence and quality of life. Although advances in AD biology have clarified major pathogenic processes, treatments that prevent or reverse disease progression remain limited. Therefore, a better understanding of early disease mechanisms is needed to guide interventions before circuit dysfunction becomes widespread (Long and Holtzman, 2019).

The traditional AD framework prioritizes the accumulation of beta-amyloid and the presence of abnormal tau pathology, a molecular perspective that has significantly influenced the development of biomarkers and therapeutic research (Selkoe and Hardy, 2016). Nevertheless, molecular pathology alone is insufficient to comprehensively account for early cognitive impairment or the variability in clinical symptoms observed in AD (De Strooper and Karran, 2016).

These observations have established neural circuit dysfunction as a valuable framework for comprehending the onset and progression of AD. Evidence derived from human imaging studies, animal models, and specific electrophysiological experiments suggests that several long-range and regulatory systems may be susceptible in the early stages of AD, although the degree of evidential directness varies across different circuits and measurement types. These systems include cortico–hippocampal, hippocampal–prefrontal, neuromodulatory, and circadian networks (Cunliffe et al., 2025; Falcicchia et al., 2023; Geula, 1998). In some cases, circuit impairment is supported by direct measures of oscillatory activity, synchrony, or interregional coupling. In other cases, it is inferred from molecular, structural, or behavioral findings. This evidence base supports a model in which impaired communication, weakened rhythmic coordination, and reduced plasticity-permissive states contribute to the reduced resilience of cognitive systems during early AD (Palop et al., 2007; Singer and Alia, 2022; Stam et al., 2007).

Exercise is increasingly recognized as a non-pharmacological intervention associated with benefits for cognition, neural plasticity, and brain network function. Epidemiological studies and clinical trials link regular physical activity to lower AD risk or slower cognitive decline, whereas animal studies provide more direct experimental support for effects on learning, plasticity, inflammation, amyloid burden, and neurotrophic signaling (Erickson et al., 2011; Sabia et al., 2017; van Praag et al., 2005). Human neuroimaging studies further indicate that physical exercise can modulate functional brain network connectivity in older adults, both with and without cognitive impairment, although earlier research primarily interpreted these benefits through molecular and cellular mechanisms, such as reduced amyloid burden, decreased inflammation, and enhanced neurotrophic support. While these mechanisms are significant, they do not fully explain the broad, system-level cognitive and behavioral changes associated with exercise (Cotman and Berchtold, 2002; Voss et al., 2011). Recent work on AD models has begun to explore exercise-sensitive circuit mechanisms, including medial septal cholinergic pathways; however, these findings remain circuit-specific and do not provide a unified account of exercise-related network modulation (Feng et al., 2025). Consequently, current evidence remains fragmented across distinct circuit systems, and a comprehensive framework for interpreting exercise-related circuit effects in early AD is still lacking.

It is crucial to recognize that exercise should not be perceived merely as a means to enhance neural activity. Available evidence supports a context-dependent model in which physical activity influences network excitability, synaptic plasticity, neuromodulatory tone, and rhythmic organization (Cotman and Berchtold, 2002; Voss et al., 2011). In early AD, the broad anatomical architecture of many circuits may remain relatively preserved, while functional stability is already compromised. Under these conditions, exercise may influence disease-relevant molecular, structural, behavioral, and network-level processes. Therefore, the central question is not whether exercise has a single circuit target, but how exercise-responsive molecular, synaptic, neuromodulatory, vascular-metabolic, and rhythmic mechanisms are expressed across AD-vulnerable circuits. In this review, we synthesize evidence relevant to four vulnerable neural systems and distinguish direct circuit-level findings from indirect support. To organize the heterogeneous literature on exercise and early AD, this review focuses on four circuit systems that represent complementary levels of neural regulation: memory-related cortical input gating, hippocampal–prefrontal integration, cholinergic modulation of hippocampal plasticity, and circadian-neuroendocrine timing (Buzsáki, 2002; Euston et al., 2012; Hasselmo and Sarter, 2011; Musiek et al., 2015; Yassa and Stark, 2011). These systems were selected because they show early vulnerability in AD, map onto cognitive or behavioral domains responsive to physical activity, and can be linked to exercise-sensitive mechanisms, such as excitability regulation, synaptic plasticity, neuromodulatory tone, and rhythmic organization (Cotman and Berchtold, 2002; Palop and Mucke, 2016; Stam et al., 2007; Voss et al., 2011). Other AD-relevant systems, including the locus coeruleus, CA3, basolateral amygdala, and default mode network, are important interacting components but are treated here as related systems rather than primary organizing axes.

### 1.1 Review scope and search strategy

**Figure 1:**
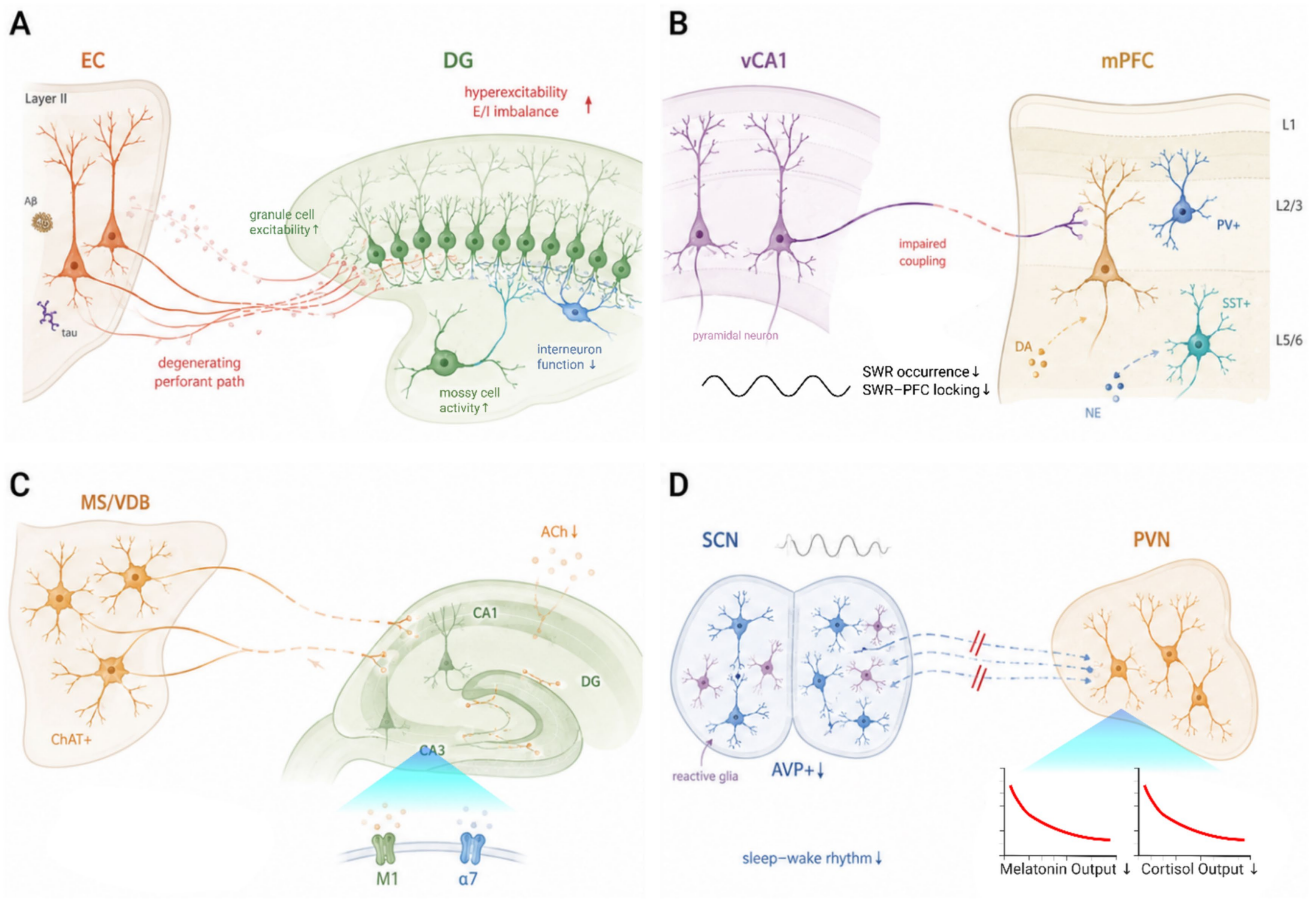
Functional vulnerability of key neural circuits in early Alzheimer’s disease. Schematic representation of four major neural circuits that exhibit early functional vulnerability in AD. **(A)** In the entorhinal cortex–dentate gyrus (EC–DG) circuit, degeneration of layer II excitatory neurons and perforant path deafferentation are associated with compensatory hyperexcitability in the DG, characterized by excitation–inhibition imbalance, network hypersynchrony, increased mossy cell activity, elevated granule cell excitability, and reduced interneuron function. **(B)** In the ventral hippocampus–medial prefrontal cortex (vHPC–mPFC) pathway, particularly ventral hippocampal CA1 projections to the mPFC, AD-related changes include impaired hippocampal input to the mPFC, disrupted hippocampal or entorhinal–hippocampal ripple coordination, excitation–inhibition imbalance, and weakened neuromodulatory regulation, collectively suggesting reduced efficiency of hippocampal–prefrontal communication. **(C)** In the medial septum/vertical diagonal band (MS/VDB)–hippocampal cholinergic circuit, degeneration of ChAT-positive cholinergic neurons leads to reduced acetylcholine availability, weakened cholinergic tone, reduced plasticity permissiveness, impaired theta–gamma coordination, and reduced synaptic plasticity. **(D)** In the suprachiasmatic nucleus–paraventricular nucleus (SCN–PVN) circadian axis, loss of AVP-positive neurons and reactive gliosis are associated with reduced sleep–wake rhythmicity, circadian phase drift, metabolic stress, neuroinflammatory vulnerability, and blunted melatonin and cortisol output. Together, these alterations summarize evidence-supported and inferred circuit vulnerabilities across memory, executive, neuromodulatory, and circadian systems in early AD. Source: BioRender.com.

This article is a narrative review. For operational purposes, we use “early AD” to denote the stage of the AD continuum in which AD-related molecular pathology and circuit dysfunction are present, but widespread neuronal loss and moderate-to-severe dementia are not yet dominant (Jack et al., 2024; Jack et al., 2018). In human studies, this includes preclinical biomarker-positive AD when relevant, prodromal AD or amnestic mild cognitive impairment, and mild AD dementia. In animal research, the term refers to model-specific pre-symptomatic or early symptomatic phases, including pre-plaque or early-plaque stages prior to extensive neurodegeneration or advanced behavioral impairment. To delineate the scope of this review, we conducted structured literature searches using PubMed, Web of Science, Google Scholar, and the reference lists of pertinent articles. The searches concentrated on combinations of terms associated with Alzheimer’s disease and its early stages (“Alzheimer’s disease,” “early Alzheimer’s disease,” “mild cognitive impairment,” “prodromal Alzheimer’s disease”), exercise or physical activity (“exercise,” “aerobic exercise,” “treadmill exercise,” “voluntary running,” “physical activity”), and circuit-specific concepts (“entorhinal cortex,” “dentate gyrus,” “perforant path,” “ventral hippocampus,” “medial prefrontal cortex,” “medial septum,” “vertical diagonal band,” “cholinergic,” “suprachiasmatic nucleus,” “paraventricular nucleus,” “circadian rhythm”). We prioritized peer-reviewed studies involving humans, animal models, and mechanistic investigations that addressed circuit vulnerability, exercise-related neural effects, or translational biomarkers pertinent to the four selected systems. Studies were included if they provided direct circuit-level evidence, electrophysiological or imaging data, or indirect molecular, structural, neurochemical, behavioral, or clinical-proxy evidence relevant to the review framework (see Figure 1).

## 2 Functional vulnerability of neural circuits in early Alzheimer’s disease

### 2.1 Disrupted cortical input gating in the EC–DG circuit

The EC–DG circuit is among the earliest and most vulnerable long-range pathways affected in AD. Layer II excitatory neurons in the entorhinal cortex provide the major excitatory input to dentate gyrus (DG) granule cells through the perforant path, a key anatomical substrate for cortical–hippocampal information transfer (Witter et al., 2000). Neuropathological and neuroanatomical studies have consistently shown marked selective vulnerability in this circuit during early AD, with structural degeneration of EC layer II neurons and the perforant pathway emerging before widespread hippocampal degeneration becomes evident (Braak and Braak, 1991; Gómez-Isla et al., 1996; Hyman et al., 1984).

Importantly, EC–DG dysfunction is not simply defined by reduced cortical input. It combines upstream deafferentation with downstream imbalances in local DG network states. EC layer II excitatory neurons are highly susceptible to AD pathology, and their degeneration is accompanied by perforant-path disruption and white-matter microstructural abnormalities detectable *in vivo* (Braak and Braak, 1991; Gómez-Isla et al., 1996; Hyman et al., 1984; Salat et al., 2010; Yassa et al., 2010). In the outer molecular layer of the DG, reduced synaptic density and decreased expression of synaptic proteins have been linked to cognitive impairment (Haytural et al., 2021; Robinson et al., 2014). When considered alongside electrophysiological evidence of DG hyperexcitability described below, these structural and synaptic findings support a circuit-level model in which EC–DG input gating becomes vulnerable early in AD and contributes to memory dysfunction.

Persistent weakening of cortical input can leave the DG in a long-term deafferented state associated with circuit instability and compensatory remodeling (Hyman et al., 1986; Palop and Mucke, 2016). Rather than becoming simply hypoactive, the DG undergoes pathological reorganization, including increased intrinsic excitability, altered synaptic integration, and abnormal firing patterns, such that input processing becomes less accurate whereas output excitation increases (Palop et al., 2007; Palop and Mucke, 2016). In AD models, disordered firing in EC layer II, weakened inhibitory control, increased mossy-cell activity, and insufficient interneuron recruitment converge to disrupt excitation–inhibition balance along the EC–DG axis (Booth et al., 2016; Busche et al., 2008; Chen L. et al., 2023; Palop et al., 2007; Palop and Mucke, 2016). This state makes the DG more prone to hypersynchronous, high-frequency activity resembling low-threshold epileptiform states, which may impair pattern separation and reduce the accuracy of new memory encoding (Bakker et al., 2012; Busche and Konnerth, 2015; Yassa and Stark, 2011).

Synaptic, molecular, and neuromodulatory disturbances may further amplify this instability. Amyloid-*β* and tau pathologies can impair glutamatergic transmission and synaptic integration in AD-relevant hippocampal circuits, providing molecular and synaptic mechanisms that may worsen EC–DG input processing (Hsieh et al., 2006; Sheng et al., 2012). In addition, GABAergic and cholinergic regulatory influences arising from local interneuron networks and the medial septum are progressively impaired in AD, a neurochemical and circuit-modulatory change that may weaken rhythmic control of the DG and reduce the permissiveness of synaptic plasticity (Haam and Yakel, 2017; Hasselmo and Sarter, 2011; Palop and Mucke, 2016; Schliebs and Arendt, 2011). Thus, molecular and neurochemical alterations are integrated into the EC–DG circuit model by shaping the timing, gain, and plasticity of cortical input.

As a circuit-state model, current evidence indicates that EC–DG dysfunction in early AD causes structural deafferentation of the pathway from EC layer II neurons to the DG, with electrophysiological evidence of compensatory hyperexcitability within the local DG network. This pathological state combines impaired input gating with unstable output dynamics, providing a circuit-level framework for early memory impairment and identifying EC–DG excitability balance as a potential intervention target. Structural, molecular, and neurochemical markers are therefore best interpreted as mechanistic substrates that help explain the observed circuit phenotype, while firing patterns, oscillations, LTP, and connectivity measures define the more direct circuit-level component of the model.

### 2.2 Impaired long-range functional coupling in the vHPC–mPFC pathway

Within the vHPC–mPFC pathway, ventral hippocampal CA1 pyramidal neurons project monosynaptically to the medial prefrontal cortex (mPFC) through glutamatergic long-range connections. Their axons mainly terminate in the second, third and fifth layers of the anterior cingulate cortex and other limbic cortical regions, including the anterior and subgenual cingulate (Hoover and Vertes, 2007). This pathway plays a core role in integrating hippocampal memory representations with prefrontal processes involved in executive control, strategy selection, and goal-directed behavior (Euston et al., 2012; Spellman et al., 2015). Under physiological conditions, the communication between the hippocampus and the prefrontal cortex is coordinated by temporally structured oscillatory interactions, especially theta-related coupling, which facilitates information transfer, memory-guided behavior, and adaptive decision-making (Jones and Wilson, 2005; Siapas et al., 2005).

Recent evidence from animal models suggests that functional coupling within hippocampal–prefrontal networks is compromised in AD, thereby linking altered hippocampal–prefrontal functional connectivity to executive dysfunction (Cunliffe et al., 2025). Concurrently, electrophysiological studies in AD models have demonstrated impaired sharp-wave ripple (SWR) coordination within entorhinal–hippocampal circuits, including disrupted coordination between the medial entorhinal cortex and CA1 (Funane et al., 2022). Given that SWRs provide temporal windows for hippocampal replay and memory consolidation, these findings align with the notion of compromised hippocampal output timing (Girardeau et al., 2009). Collectively, the evidence from functional connectivity and oscillatory studies supports a circuit-level interpretation of hippocampal–prefrontal dysfunction, while also clarifying that different studies have examined distinct nodes and temporal scales within the broader system.

The vHPC–mPFC pathway demonstrates synaptic vulnerability, particularly concerning the ventral hippocampal CA1 projections to the mPFC. Aβ and tau pathologies can disrupt presynaptic neurotransmitter release and postsynaptic responsiveness, offering molecular and synaptic evidence for impaired transmission and plasticity within hippocampal–prefrontal circuits (Capilla-López et al., 2025; Flores-Martínez and Peña-Ortega, 2017; Wang et al., 2025). Experimental studies, including non-AD circuit manipulation paradigms, further illustrate functionally organized hippocampal–prefrontal assemblies and causal hippocampal influences on mPFC-related behavior (Domanski et al., 2023; Yoon et al., 2024). These synaptic and molecular findings do not demonstrate hippocampal–prefrontal decoupling; however, they provide circuit-relevant context for how AD pathology could weaken hippocampal input to prefrontal networks.

Disruption of this pathway is compounded by local mPFC inhibitory and neuromodulatory deficits. AD-related dysfunction of PV + and SST + interneurons may weaken temporal control of excitatory inputs and exacerbate local excitatory-inhibitory imbalance (Shu et al., 2023). The degeneration of noradrenergic and dopaminergic inputs may further reduce state-dependent regulation of hippocampal signals by the mPFC, limiting circuit flexibility under varying behavioral demands (Dahl et al., 2023). These cellular and neurochemical findings identify mechanisms through which local mPFC state control can destabilize long-range coordination, complementing the direct functional connectivity and oscillatory evidence above.

AD-related hippocampal–prefrontal impairment is characterized by altered functional connectivity and neural dynamics, weaker synaptic transmission, and deficits in inhibitory and neuromodulatory regulation. This long-range circuit decoupling may limit the transfer of memory-related information to executive networks, contributing to impairments in executive functioning, decision-making flexibility, and contextual adaptation.

### 2.3 Degeneration of cholinergic modulatory control in the MS/VDB–hippocampal circuit

Cholinergic neurons in the medial septum (MS) and vertical diagonal band (VDB) are major neuromodulatory sources for the hippocampus (He et al., 2023). Through ChAT+ cholinergic projections, this system influences multiple hippocampal subregions by regulating theta and gamma oscillations, promoting synaptic plasticity, supporting attention, and facilitating memory encoding (Moghadam et al., 2025; Pirazzini and Ursino, 2025). Under normal conditions, the MS/VDB–hippocampal cholinergic circuit helps maintain a hippocampal state that is permissive for plasticity (Kopsick et al., 2022).

Evidence from human studies and animal models shows clear functional impairment of this cholinergic system in early AD. Brain tissue analyses and experimental studies report reduced choline acetyltransferase-positive (ChAT+) neurons, loss of cholinergic nerve fibers projecting to the hippocampus, and progressive decreases in acetylcholine (ACh) levels (Scharfman et al., 2025; Wu et al., 2023). Because this damage can occur before major neuronal loss in the hippocampus and cortex, cholinergic dysfunction is best framed as a potential contributor to early network abnormalities, not only as a secondary consequence of neuronal injury (Schmitz and Nathan Spreng, 2016).

The loss of cholinergic input can impair the stability and temporal control of rhythmic hippocampal activity. Reduced theta strength, gamma power, and interregional rhythm synchrony are frequently observed in AD models; such oscillatory impairment can weaken temporal integration and affect memory encoding and temporal-order processing (Berg et al., 2025). Conversely, optogenetic enhancement of medial septal cholinergic input to the hippocampus can partially restore theta–gamma coupling and improve synaptic plasticity (Gu and Yakel, 2022; Vandecasteele et al., 2014).

Cholinergic signaling is further weakened by impaired receptor-level responsiveness in AD. Functional deficits in M1 muscarinic receptors (M1 mAChR) and alpha7 nicotinic acetylcholine receptors (alpha7 nAChR) have been reported, which may increase the threshold for long-term potentiation (LTP) induction, reduce neuronal responsiveness to afferent input, and limit learning-related synaptic plasticity (Rennie, 2025; Shinoe et al., 2005). Degeneration also affects non-cholinergic medial septal neurons, including GABAergic PV + cells that contribute to hippocampal rhythm generation and timing control, thereby further weakening oscillatory regulation in the hippocampal network (Király et al., 2023; Leitch, 2024).

The MS/VDB–hippocampal circuit exhibits a significant functional decline in AD, characterized by degeneration of cholinergic neurons, diminished neurotransmitter availability, reduced receptor responsiveness, and disrupted rhythmic control of hippocampal activity. At the circuit level, this pattern leads to decreased synaptic plasticity, impaired hippocampal memory encoding, and disrupted temporal coordination, which are essential for learning and memory. In the early stages of AD, cholinergic dysfunction is not merely a reduction in neurotransmitter levels but rather a loss of circuit-level regulation and rhythmic coordination crucial for hippocampus-dependent cognition.

### 2.4 Circadian network dysregulation along the SCN–PVN circuit

The suprachiasmatic nucleus (SCN) serves as the primary circadian pacemaker in mammals, orchestrating diurnal rhythms and transmitting temporal cues to the preoptic area (POA) and other hypothalamic regions responsible for regulating hormonal rhythms, including those of melatonin and cortisol. Through these signaling pathways, the SCN plays a crucial role in the organization of sleep–wake cycles, energy metabolism, and the maintenance of endocrine homeostasis (Ma and Morrison, 2025). Clinical studies consistently show disrupted sleep–wake cycles, weakened daily activity rhythms, and phase shifts in individuals with AD, a pattern consistent with dysfunction of the SCN and its output pathways even at early disease stages (Musiek et al., 2015; Sharma et al., 2021). These clinical observations should therefore be viewed not only as sleep-quality symptoms, but also as indirect evidence of disturbed central clock output and circadian organization (Musiek et al., 2015; Sharma et al., 2021).

Existing evidence indicates that the SCN network undergoes coordinated structural and functional degeneration in AD (Singer and Alia, 2022). Human autopsy and animal model studies have reported reduced SCN volume, decreased numbers of clock neurons, and prominent loss of arginine vasopressin (AVP) positive neurons (Liu et al., 2000; Zhou et al., 2016). Increased glial cell proportions further suggest that neurodegeneration and local inflammatory responses may act together (Son et al., 2024). These structural and cellular changes provide an anatomical basis weakened coupling among clock neurons and for reduced amplitude and stability of rhythmic output.

Circadian firing differences among SCN neurons are significantly weakened in AD models, and rhythmic activity becomes flattened and unstable (Paul et al., 2018). This electrophysiological evidence supports the view that AD pathology can degrade the temporal coding needed for SCN output to downstream targets, including the PVN. Consistent with this view, clinical studies have shown reduced circadian amplitudes of melatonin and cortisol in AD, with rhythm flattening closely associated with sleep fragmentation, phase drift, and metabolic disturbance (Cardinali et al., 2010; Weldemichael and Grossberg, 2010). These endocrine rhythm disorders are not only manifestations of sleep disturbance but may also disrupt brain functional homeostasis.

GABAergic signaling dominates in the SCN and is crucial for phase coordination among SCN neurons and maintenance of network synchronization (DeWoskin et al., 2015). AD-associated disturbances in GABAergic signaling may therefore reduce the accuracy of population-level rhythm coordination within the SCN and impair circadian output (Ahmad et al., 2023). Melatonin-mediated feedback regulation may also be affected: MT1 receptors show circadian-time-dependent localization in the rat SCN (Waly and Hallworth, 2015), and Aβ can impair melatonin synthesis and melatonin receptor signaling in experimental systems (Cecon et al., 2015). The subtype-specific vulnerability of AVP-positive neurons may further compromise circadian timing and phase stability (Liu et al., 2000).

Overall, the SCN–PVN circuit in AD exhibits multilevel impairment, including selective loss of clock neurons, weakened rhythmic firing, and desynchronization of neuroendocrine output. Disruption of this system contributes to sleep–wake abnormalities and may exacerbate neuroinflammation, metabolic stress, and neurodegeneration through persistent circadian imbalances. Circadian rhythm dysfunction in AD should therefore be framed not merely as a behavioral or endocrine symptom, but as a circuit-level instability that may feed back onto cognitive decline and disease progression.

**Figure 2:**
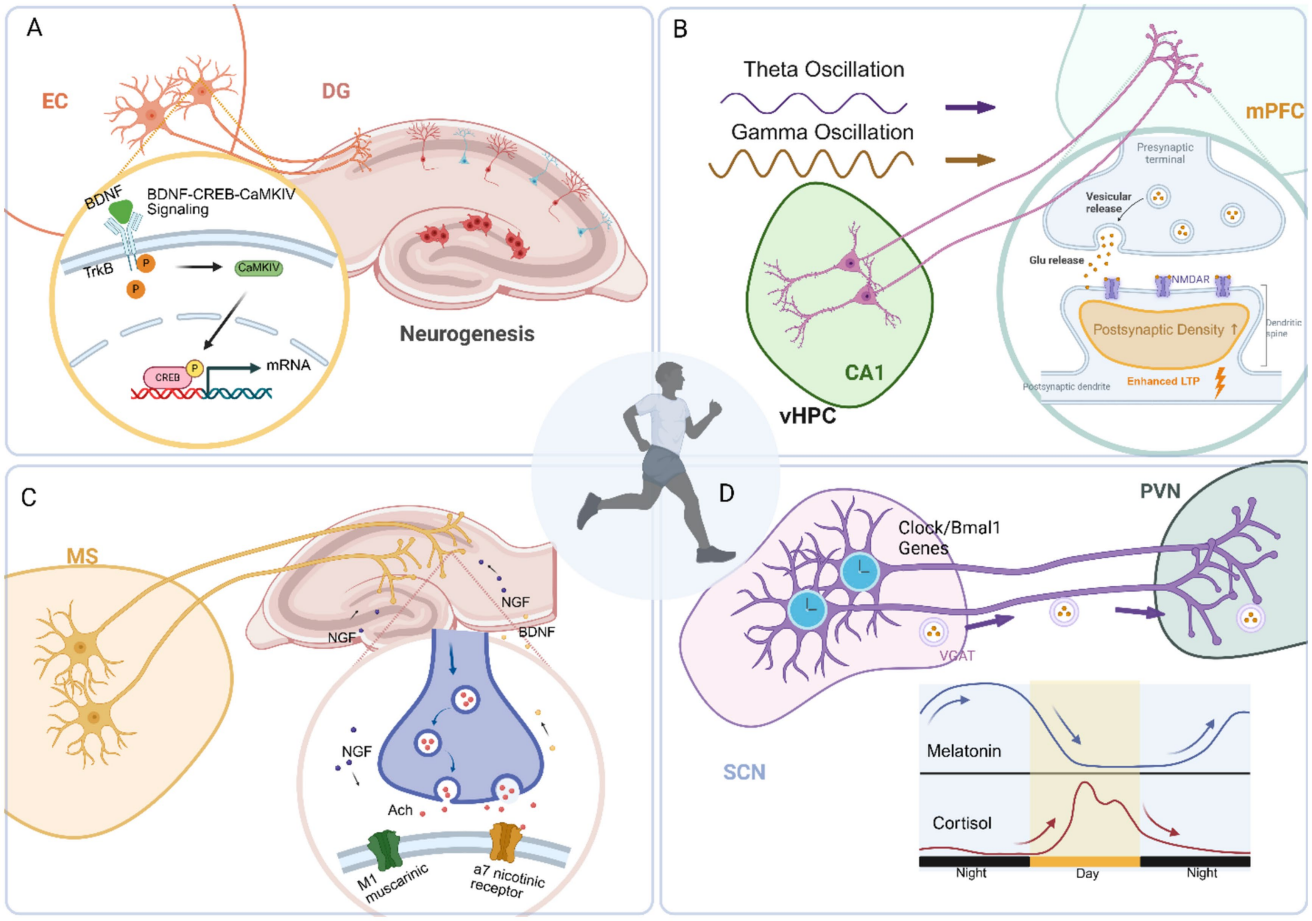
Exercise-mediated remodeling of vulnerable neural circuits in early Alzheimer’s disease. The schematic representation delineates a multicircuit framework that connects exercise-responsive mechanisms to neural systems susceptible to AD These mechanisms encompass excitability regulation, plasticity, neuromodulation, vascular-metabolic support, and circadian-neuroendocrine timing. **(A)** Within the EC–DG circuit, exercise may enhance the signaling pathways of BDNF–CREB–CaMKIV, support perforant path input, promote adult neurogenesis, and contribute to the maintenance of the structural and functional stability of the dentate gyrus circuitry. **(B)** In the vHPC–mPFC pathway, the effects sensitive to exercise are most effectively represented as support for hippocampal and prefrontal plasticity, which includes long-term potentiation, postsynaptic density, synaptic density, dendritic complexity, and hippocampal functional connectivity. **(C)** Within the MS/VDB–hippocampal cholinergic circuit, exercise may upregulate neurotrophic support, including NGF and BDNF, preserve cholinergic signaling, and support ACh-mediated modulation through mechanisms associated with muscarinic and nicotinic receptors. **(D)** In the SCN–PVN circadian axis, exercise may reinforce clock-gene rhythmicity, including Clock/Bmal1-related oscillatory regulation, preserve GABAergic output and VGAT-associated synaptic function, and improve endocrine rhythmicity, including melatonin and cortisol dynamics. Collectively, the figure links each exercise-responsive mechanism to a specific vulnerable circuit, while distinguishing supported component-level effects from circuit-level hypotheses that require direct testing in early AD. Source: BioRender.com.

Translationally, however, the SCN–PVN axis remains difficult to assess directly in living patients. Human evidence for SCN involvement comes mainly from postmortem anatomy, limited neuroimaging-oriented approaches, rest-activity recordings, melatonin or cortisol profiles, body temperature, and other proxy measures (Dijk and Duffy, 2020; Hu et al., 2013; Singer and Alia, 2022; Swaab et al., 1985). These tools are valuable for circadian phenotyping, and actigraphy-derived rhythm fragmentation has been linked to cognitive decline in mild-to-moderate AD (Targa et al., 2021). Nevertheless, they do not localize dysfunction to the SCN–PVN pathway with the same precision as rodent electrophysiology, targeted recording, or circuit manipulation. The clinical interpretation of circadian disruption should therefore distinguish observable rhythm instability from direct measurement of SCN–PVN circuit failure (Figure 2).

## 3 Exercise-related modulation of neural circuit states in early Alzheimer’s disease

The exercise literature reviewed here spans controlled treadmill training, voluntary wheel running, walking, Tai Chi, timed exercise, and combined aerobic–resistance paradigms, reflecting the diversity of exercise protocols used in animal and human studies of AD-related brain outcomes (De Sousa et al., 2021). While these interventions vary in terms of intensity, duration, frequency, timing, and experimental control, they converge on a common principle: repeated physical activity can activate adaptive biological pathways that manifest differently across circuits vulnerable to AD (Cotman and Berchtold, 2002; Voss et al., 2011). These processes encompass excitability regulation, synaptic plasticity, neurotrophic support, cholinergic modulation, vascular and metabolic enhancement, and the stabilization of behavioral or endocrine rhythms. The subsequent sections explore how this shared exercise-responsive biology is expressed across the EC–DG, vHPC–mPFC, MS/VDB–hippocampal, and SCN–PVN circuits.

### 3.1 Exercise-related modulation of EC–DG excitability balance and dentate gyrus plasticity

In early AD, the perforant pathway linking entorhinal cortex layer II neurons with the DG often shows a disinhibition-hyperexcitation pattern, in which cortical input is weakened, whereas DG excitability is excessively enhanced. Against this backdrop, exercise-related benefits are best interpreted as circuit-relevant modulation of excitability balance and plasticity-related processes within the EC–DG axis, rather than as a simple increase in neural activity.

At the upstream cortical-drive level, long-term moderate aerobic treadmill exercise (1 h/day, 5 days/week, 16 weeks) can normalize membrane excitability in EC excitatory neurons of 3xTg-AD mice and rebalance excitatory and inhibitory synaptic transmission (Chen H. et al., 2023). Consistent with this interpretation, moderate treadmill exercise (5 days/week, 4 weeks) prevented DG long-term potentiation impairment and restored plasticity-related signaling pathways in an Aβ-induced rat model (Dao et al., 2015). These electrophysiological and synaptic-plasticity findings support a circuit-relevant interpretation of exercise effects on EC–DG state regulation, while interregional synchrony and phase coordination remain endpoints for future direct testing.

Molecular evidence further supports the biological substrates through which exercise could create a plasticity-permissive EC–DG state. In hippocampal and DG models, exercise has been associated with the upregulation of brain-derived neurotrophic factor (BDNF) and the activation of cAMP response element-binding protein (CREB) and calcium/calmodulin-dependent protein kinase IV (CaMKIV)-related signaling pathways (Dao et al., 2015; Zagaar et al., 2013). These pathways are crucial for the maintenance of long-term potentiation (LTP), synaptic strengthening, and activity-dependent plasticity. These DG-related molecular changes provide a mechanistic basis for a plasticity-permissive environment in which granule-cell circuits may better maintain synaptic responsiveness to cortical input.

Voluntary wheel-running paradigms, which typically involve unrestricted access to running over several weeks with self-determined distance and intensity, have been shown to enhance adult hippocampal neurogenesis, particularly within the DG, thereby providing structural evidence for a potential substrate of long-term microcircuit remodeling (Gonçalves et al., 2016; van Praag et al., 1999). Voluntary running paradigms (1 month) further indicate that exercise not only increases the number of new neurons but also remodels their afferent connections (Vivar et al., 2016). These structural changes may facilitate sparse coding and more efficient integration of cortical inputs. Within the circuit-state model, these findings align with a dual mechanism whereby exercise supports entorhinal cortex (EC)–DG function through both acute physiological normalization and sustained remodeling of the DG microcircuit structure.

Existing evidence predominantly supports a model wherein exercise enhances specific components pertinent to EC–DG function, including medial entorhinal excitability, DG synaptic plasticity, plasticity-related molecular signaling, and adult neurogenesis. Consequently, the evidence base encompasses cellular electrophysiology, synaptic plasticity, molecular signaling, and structural remodeling, supporting the interpretation that exercise may shift the components of the EC–DG axis towards a more stable and plasticity-permissive state. Future circuit-level recordings or activity-imaging studies should further investigate how these component-level effects translate into EC–DG input-gating dynamics during memory behavior.

### 3.2 Exercise-sensitive plasticity relevant to vHPC–mPFC communication

The vHPC–mPFC pathway provides a circuit interface through which hippocampal memory representations can influence prefrontal processes involved in working memory, executive control, decision-making, and behavioral flexibility. Under physiological conditions, hippocampal–prefrontal communication depends on temporally organized neural activity, including hippocampal–prefrontal theta coupling, sharp-wave ripple-associated coordination, and the integrity of ventral hippocampal input to the mPFC (Euston et al., 2012; Funane et al., 2022; Spellman et al., 2015). AD model studies increasingly indicate that this circuit is vulnerable during disease progression. In 3xTg-AD mice, executive dysfunction is associated with altered hippocampal–prefrontal functional connectivity and impaired short-term synaptic plasticity at hippocampal inputs to the mPFC (Cunliffe et al., 2025). In APP/PS1 mice, simultaneous vHPC and mPFC recordings show reduced theta and low-gamma directed information flow during working-memory encoding (Ai et al., 2025). In 5xFAD mice, disrupted hippocampal–prefrontal neural dynamics, impaired sharp-wave ripple-associated corticolimbic coordination, and diminished hippocampal–prefrontal synchrony accompany deficits in adaptive decision making (Kim et al., 2025). Together, these studies identify hippocampal–prefrontal communication as a circuit-level substrate for the executive and working memory deficits that accompany AD pathology.

Exercise is relevant to this pathway because it targets several biological requirements for effective hippocampal–prefrontal communication. In APP/PS1 mice, long-term aerobic treadmill exercise (30 min/day, 5 days/week, 5 months) improves spatial learning and memory together with hippocampal long-term potentiation, indicating preservation of synaptic function in a memory-related circuit node (Liu et al., 2011). In 3xTg-AD mice, aerobic treadmill training (1 h/day, 5 days/week, 12 weeks) improves spatial working memory and enhances structural synaptic plasticity in both the hippocampus and prefrontal cortex, including increased synaptic density, thicker postsynaptic density, and greater dendritic complexity (Mu et al., 2022). These findings are important for the vHPC–mPFC framework because long-range coordination depends not only on oscillatory timing but also on the synaptic efficacy, dendritic architecture, and plasticity of the connected hippocampal and prefrontal nodes.

Human neuroimaging and EEG studies provide an important translational bridge between rodent circuit mechanisms and clinical early-AD populations. In mild cognitive impairment (MCI), 12 weeks of walking exercise increased PCC/precuneus resting-state connectivity within the default-mode network (Chirles et al., 2017), and a separate 12-week exercise intervention increased anterior and posterior hippocampal connectivity, with connectivity changes related to memory performance (Won et al., 2021). In the SYNERGIC trial, exercise alone or combined with cognitive training and vitamin D3 increased functional brain connectivity within default-mode network regions in older adults with MCI, including hippocampal–angular gyrus connectivity (Bray et al., 2023). EEG-focused evidence further shows exercise-related modulation of cortical activity in MCI, including changes in delta/theta power, alpha/beta activity, EEG complexity, and P300 measures (Huang et al., 2022; Pedroso et al., 2021). Together, these human data support the use of resting-state fMRI and EEG/ERP endpoints as translational readouts for testing whether exercise stabilizes AD-relevant network states.

For the vHPC–mPFC pathway, the strongest defensible conclusion is that exercise supports the synaptic, structural, neurotrophic, and largescale network conditions required for hippocampal–prefrontal communication. This model links AD-related vHPC–mPFC circuit vulnerability with exercise sensitive plasticity in hippocampal and prefrontal systems, while preserving the key experimental question for the field: whether defined exercise paradigms can improve vHPC–mPFC synchrony, coherence, or directional information flow in AD models during executive or working memory behavior. Addressing this question will require simultaneous vHPC–mPFC recordings, activity imaging, or causal circuit manipulation during exercise intervention studies.

### 3.3 Exercise-related modulation of MS/VDB–hippocampal cholinergic function

During early AD, basal forebrain cholinergic dysfunction represents a major neuromodulatory disturbance that can compromise hippocampal circuit regulation. The medial septum/vertical diagonal band–hippocampal (MS/VDB–hippocampal) projection is particularly relevant because septal cholinergic input regulates hippocampal acetylcholine availability, theta dynamics, plasticity-permissive states, and memory encoding. AD-related studies indicate that MS/VDB–hippocampal cholinergic vulnerability contributes to impaired memory consolidation and hippocampal network dysfunction (Schmitz and Nathan Spreng, 2016; Wu et al., 2023). Thus, cholinergic dysfunction in early AD should be viewed not only as reduced transmitter availability, but also as a loss of neuromodulatory control over hippocampal excitability, rhythmic coordination, and synaptic plasticity.

Voluntary wheel-running paradigms provide direct evidence in non-AD cholinergic-deficiency models that exercise can recruit septohippocampal, or MS/VDB–hippocampal, cholinergic plasticity under conditions of cholinergic vulnerability. In a cholinergic-deficiency model, voluntary wheel running (2 weeks) rescued behaviorally stimulated hippocampal acetylcholine (ACh) efflux, promoted re-emergence of a cholinergic/nestin phenotype within the medial septum/diagonal band, and improved spatial working memory (Hall and Savage, 2016). Subsequent work further showed that nerve growth factor (NGF) is required for voluntary running (2 weeks) induced recovery of septohippocampal cholinergic structure and function (Hall et al., 2018). These studies are not AD models, but they provide direct evidence that exercise can recruit septal cholinergic plasticity and restore hippocampal ACh-related function.

Aerobic activity paradigms can also support the hippocampal environment in which cholinergic modulation operates. In APP/PS1 mice, long-term aerobic treadmill exercise (30 min/day, 5 days/week, 5 months) improved hippocampal long-term potentiation and memory performance, indicating exercise-related preservation of hippocampal plasticity in an AD model (Liu et al., 2011). In aged mice, long-term voluntary running (1 h/day, 5 days/week, 5 months), performed over several months in late life, improved spatial memory and preserved cholinergic inputs in hippocampal CA1 and DG, supporting a link between voluntary exercise, hippocampal cholinergic innervation, and memory-related function (Xu et al., 2019). Short-term voluntary exercise (1 week)-induced BDNF-related signaling may further contribute to plasticity-permissive hippocampal states (Vaynman et al., 2004). Because septohippocampal cholinergic input is a major regulator of hippocampal theta dynamics and learning-related plasticity, these findings are consistent with the possibility that exercise supports hippocampal function partly by maintaining a cholinergic-compatible network environment (Buzsáki, 2002; Lee et al., 2005).

AD-model evidence is beginning to connect exercise with medial septal cholinergic circuitry, although not always through the same projection emphasized in the septohippocampal framework. Feng et al. reported that moderate-intensity treadmill exercise (1 h/day, 3 days/week, 5 weeks) combined with resistance ladder climbing exercise (27 climbs/day, 5 weeks) improved cognitive outcomes in 5 × FAD mice through a medial septal–medial habenula cholinergic circuit (Feng et al., 2025). This finding provides AD-specific support for the broader idea that medial septal cholinergic networks can be recruited by exercise and can contribute to cognitive benefits. In this sense, the study extends the cholinergic exercise framework from non-AD septohippocampal recovery models toward AD-relevant medial septal circuitry.

The available evidence supports a model in which exercise may help maintain cholinergic regulatory control over hippocampal function through neurotrophic, neurochemical, structural, and oscillatory mechanisms. The strongest exercise evidence for septohippocampal cholinergic recovery comes from cholinergic-deficiency and aging models, whereas AD-specific exercise evidence currently implicates a related medial septal cholinergic circuit rather than directly demonstrating restoration of the MS/VDB–hippocampal projection. Accordingly, the MS/VDB–hippocampal component of the framework can best be interpreted as a convergent cholinergic mechanism, supported by exercise-related recovery of septohippocampal function in cholinergic-deficiency models, hippocampal plasticity findings in AD models, and emerging AD-specific evidence from related medial septal circuitry.

### 3.4 Exercise-related modulation of circadian output and neuroendocrine rhythms relevant to the SCN–PVN axis

In early AD, weakening of SCN output may contribute to circadian disruption and downstream neuroendocrine imbalance (Ono et al., 2021). Physical activity can be framed as a non-photic circadian modulator that strengthens behavioral rhythmicity, sleep organization, and endocrine-related timing signals in AD models. Within the present framework, these changes are interpreted as circuit-relevant evidence for improved SCN–PVN-related output regulation, while full circuit-level rescue of the SCN–PVN axis remains an evidence-informed hypothesis (Jones et al., 2021; Sasaki et al., 2016).

Chronic voluntary wheel running exercise has been reported to improve circadian and sleep-related abnormalities in AD models. In APP/PS1 mice, voluntary wheel running (2 months) significantly improved behavioral circadian rhythm disturbance and sleep structure, with these behavioral changes occurring alongside partial recovery of circadian endocrine-related measures (Hu et al., 2025). This pattern is consistent with voluntary running-related reintegration of behavioral and neuroendocrine rhythms under AD conditions. More broadly, forced or voluntary running paradigms can act as non-photic circadian modulators capable of shifting behavioral and physiological phase relationships and influencing clock-controlled rhythmic outputs (Sasaki et al., 2016; Yasumoto et al., 2015). Timed exercise can influence circadian timing mechanisms and improve rhythm robustness, but current studies do not uniformly support largescale restoration of SCN molecular programs. For example, scheduled voluntary exercise (6 h/day, 3 weeks) has been shown to stabilize behavioral rhythms without broadly normalizing the dysregulated SCN transcriptome, suggesting that behavioral circadian rescue can occur even in the absence of major SCN-wide molecular reprogramming (Hitrec et al., 2023). Accordingly, exercise is best interpreted as strengthening circadian output and phase organization through selected behavioral and cellular mechanisms, rather than fully restoring SCN clock-gene regulation (Hitrec et al., 2023; Yasumoto et al., 2015).

Increased direct support for structural and molecular protection of SCN GABAergic neurons. In APP/PS1 mice, voluntary wheel running reduced tau phosphorylation and axonal damage in SCN GABAergic neurons and increased vesicular GABA transporter (VGAT) expression, changes consistent with improved inhibitory output capacity from the SCN (Hitrec et al., 2023; Hu et al., 2025). Given the importance of GABAergic signaling for synchrony and temporal precision within the SCN network, this evidence aligns with the possibility that exercise enhances circadian output stability by preserving SCN inhibitory circuitry (Hu et al., 2025; Ono et al., 2021).

Because PVN circadian neurons are required to sustain daily glucocorticoid rhythms, improved SCN output would be expected to favor more stable downstream neuroendocrine coordination (Jones et al., 2021). Even so, direct evidence for exercise-mediated rescue of the full SCN–PVN circuit in AD remains limited. The available data support a model in which exercise improves circadian function by stabilizing behavioral rhythms, preserving SCN GABAergic structure, and enhancing the conditions necessary for coordinated endocrine rhythmicity.

Current evidence indicates that exercise can improve circadian-relevant outcomes in AD models, including behavioral rhythmicity, sleep organization, selected SCN inhibitory-neuron markers, and endocrine-related timing measures. These outcomes are consistent with a model in which exercise may stabilize SCN–PVN-related output regulation through behavioral, cellular, molecular, and neuroendocrine mechanisms. Most mechanistic exercise studies rely on nocturnal rodents under controlled light–dark cycles, housing conditions, and wheel-access paradigms, whereas older adults with AD are diurnal and highly heterogeneous in chronotype, medication exposure, mobility, comorbid sleep disorders, and daily light exposure (Challet, 2007; Nassan and Videnovic, 2022; Smale et al., 2003). Therefore, human studies should evaluate clinically feasible circadian endpoints, such as actigraphy-derived rhythm stability, sleep timing, dim-light melatonin onset, or serial salivary melatonin/cortisol profiles, together with cognitive and behavioral outcomes (Dijk and Duffy, 2020; Targa et al., 2021). In this translational context, exercise-mediated stabilization of the SCN–PVN axis is best presented as a testable circuit model supported by convergent behavioral, cellular, molecular, and neuroendocrine evidence.

The circuit-specific evidence reviewed above is summarized in Table 1, which integrates AD-related circuit alterations, exercise-associated effects, proposed mechanisms, and the relative levels of animal and human evidence (Table 1).

**Table 1:** Summary of AD-related circuit alterations, exercise-associated effects, proposed mechanisms, and evidence levels.

Circuit	AD-related alterations	Exercise-associated effects	Proposed mechanisms	Evidence level and representative references
EC–DG	Entorhinal/perforant-path vulnerability, DG hyperexcitability, and impaired input gating.	Treadmill or voluntary running supports entorhinal excitability control, DG plasticity, and neurogenesis.	Excitability rebalance, BDNF–CREB–CaMKIV signaling, LTP support, and DG remodeling.	Mainly on exercise in animals, with human evidence supporting EC/DG vulnerability (Bakker et al., 2012; Chen H. et al., 2023; Dao et al., 2015; Vivar et al., 2016).
vHPC–mPFC	Impaired hippocampal–prefrontal coupling, disrupted timing, and synaptic and inhibitory deficits.	Treadmill exercise improves hippocampal LTP, memory, and hippocampal/prefrontal synaptic structures.	Synaptic and structural plasticity may support long-range coordination.	Animal AD model evidence supports node-level exercise effects; direct exercise rescue of vHPC–mPFC synchrony remains untested (Cunliffe et al., 2025; Kim et al., 2025; Liu et al., 2011; Mu et al., 2022).
MS/VDB–hippocampal	Cholinergic degeneration, reduced hippocampal ACh availability, and impaired theta–gamma organization.	Voluntary running restores septohippocampal ACh efflux in cholinergic-deficiency models; running preserves hippocampal cholinergic inputs in aging models; AD-specific exercise evidence implicates a related MS–MHb cholinergic circuit.	NGF/BDNF support, cholinergic signaling, hippocampal ACh modulation, and theta–gamma stabilization.	Direct animal evidence for non-AD septohippocampal recovery; AD-specific evidence for related medial septal circuitry; direct MS/VDB–hippocampal rescue in AD remains to be tested (Feng et al., 2025; Hall et al., 2018; Hall and Savage, 2016; Xu et al., 2019).
SCN–PVN	Clock neuron vulnerability, rest–activity disruption, and sleep and neuroendocrine dysrhythmia.	Voluntary or timed exercise improves behavioral rhythmicity, sleep organization, selected SCN-related markers, and endocrine timing.	Non-photic entrainment, SCN inhibitory output, and melatonin/cortisol coordination.	Mainly animal exercise evidence; human evidence relies on actigraphy, sleep, endocrine, and other proxy measures (Hitrec et al., 2023; Hu et al., 2025; Targa et al., 2021; Yasumoto et al., 2015).

Evidence levels summarize the dominant evidence types reviewed in the manuscript. The detailed exercise parameters are described in the corresponding text sections.

## 4 Discussion

This review advances a circuit-state interpretation of exercise-related benefits in early AD, building on existing integrative accounts of exercise-brain interactions. Earlier work established exercise as a behavioral intervention that promotes brain plasticity through neurotrophic, synaptic, metabolic, vascular, and cognitive mechanisms across the lifespan (Cotman and Berchtold, 2002; Raichlen and Alexander, 2017; Stillman et al., 2016; Voss et al., 2011). The present review extends this perspective by organizing these exercise-responsive processes within AD-vulnerable circuit systems. Across cortico–hippocampal, hippocampal–prefrontal, neuromodulatory, and circadian systems, exercise-sensitive changes converge on neural properties that are central to circuit stability: excitability control, synaptic plasticity, neurotrophic support, cholinergic modulation, and rhythmic organization (Chen H. et al., 2023; Feng et al., 2025; Hall and Savage, 2016; Hu et al., 2025; Liu et al., 2011; Mu et al., 2022). This circuit-state framing helps explain how broad exercise-related biological effects may support information processing, learning, and adaptation during a disease stage in which circuit architecture may remain partly preserved but circuit dynamics are already vulnerable.

The evidence follows a circuit- and paradigm-specific gradient. In the EC–DG system, treadmill and voluntary running studies support for entorhinal excitability control, DG plasticity, and adult neurogenesis (Chen H. et al., 2023; van Praag et al., 1999; Vivar et al., 2016; Zagaar et al., 2013). In hippocampal–prefrontal systems, AD model evidence identifies disrupted connectivity and neural dynamics, while exercise studies support hippocampal and prefrontal synaptic and structural plasticity as substrates for future circuit-level testing (Ai et al., 2025; Cunliffe et al., 2025; Kim et al., 2025; Liu et al., 2011; Mu et al., 2022). In MS/VDB–hippocampal systems, voluntary running and combined aerobic–resistance exercise link exercise to cholinergic and neurotrophic mechanisms that may support hippocampal responsiveness (Feng et al., 2025; Hall et al., 2018; Hall and Savage, 2016). In SCN–PVN-related systems, voluntary or timed exercise is most strongly associated with behavioral rhythmicity, SCN-related cellular markers, and neuroendocrine timing (Hitrec et al., 2023; Hu et al., 2025; Sasaki et al., 2016; Yasumoto et al., 2015). This evidence gradient clarifies why exercise-related circuit modulation is best understood as a convergent framework of network stabilization rather than a uniform effect across all circuits and endpoints.

The shared circuit-stability framework gains interpretive precision when exercise prescription is considered as a biological dose. Controlled treadmill protocols define workload, session length, frequency, and intervention duration, supporting comparisons of excitability, LTP, and synaptic structure across AD models (Chen H. et al., 2023; Liu et al., 2011; Mu et al., 2022). Voluntary wheel running captures sustained self-selected activity and links habitual movement to neurogenesis, cholinergic plasticity, and circadian rhythmicity (Hall and Savage, 2016; Hu et al., 2025; van Praag et al., 1999). Timed exercise adds a phase component that is particularly relevant to SCN-related outputs and behavioral rhythmicity (Hitrec et al., 2023; Sasaki et al., 2016; Yasumoto et al., 2015). Intensity and duration shape the magnitude and persistence of these adaptations, as shown by dose-sensitive effects of treadmill training on Aβ burden and cognition in Tg2576 mice (Moore et al., 2016; Thomas et al., 2020). The prescription variables elucidate why the effects of exercise across circuits are predominantly manifested as convergent shifts in plasticity, neuromodulatory tone, and rhythmic organization, while individual endpoints exhibit variability across different studies.

Model context is equally important for translating these findings into an early-AD framework. The evidence reviewed encompasses various models, including 3xTg-AD, APP/PS1, 5xFAD, Tg2576, Aβ-induced rat models, cholinergic-deficiency models, aging models, and human cohorts with MCI or older adults. These systems exhibit differences in dominant pathology, age of onset, inflammatory profile, cholinergic involvement, circadian phenotype, sex effects, and disease progression. For instance, 3xTg-AD mice exhibit both amyloid- and tau-related pathology, while APP/PS1 and Tg2576 models primarily focus on amyloid-driven mechanisms. In contrast, 5xFAD mice demonstrate rapid amyloid accumulation with significant inflammatory features (Jankowsky and Zheng, 2017; Zhong et al., 2024). Mapping the effects of exercise onto these model characteristics elucidates why treadmill effects on EC–DG excitability, APP/PS1 hippocampal LTP, 5xFAD medial septal circuitry, and APP/PS1 circadian outputs can be interpreted as convergent evidence for exercise-sensitive circuit regulation, while preserving the biological specificity of each model.

Null and mixed findings contribute to delineating the conditions under which exercise-responsive circuit stabilization is most probable. In APPswe/PS1dE9 mice, voluntary wheel running enhanced cognitive function and synaptic/metabolic markers when initiated in young mice, but not when commenced at a middle-aged stage with more advanced pathology (Wang et al., 2021). In female 5xFAD mice, prolonged voluntary running did not mitigate insoluble Aβ, hippocampal neuroinflammation, cytokine levels, or non-cognitive behavioral abnormalities (Svensson et al., 2020). These studies contextualize exercise responsiveness within a biologically specific framework: exercise can activate synaptic, metabolic, neuromodulatory, inflammatory, and rhythmic pathways across Alzheimer’s disease-relevant circuits; however, observable benefits are contingent upon the disease stage, model background, sex, exercise dose and timing, and the measured outcomes. Consequently, sex should be regarded as a biological modifier of both AD vulnerability and exercise responsiveness, particularly given that synaptic, inflammatory, metabolic, cholinergic, and circadian mechanisms may vary between males and females. Future research should report sex composition, analyze male and female responses separately when adequately powered, and investigate whether exercise dose, timing, and modality interact with sex to influence circuit-level outcomes.

In clinical translation, exercise should not be regarded merely as general lifestyle exposure but rather as a collection of circuit-specific intervention hypotheses associated with quantifiable biological domains. Aerobic or walking-based programs are particularly suitable for evaluating plasticity and connectivity outcomes within the EC–DG and hippocampal–prefrontal systems. Combined aerobic–resistance paradigms may effectively capture neurotrophic, metabolic, and neuromodulatory adaptations pertinent to MS/VDB–hippocampal regulation, while timed exercise offers a logical approach for investigating SCN–PVN-related circadian output. Consequently, outcome measures should be aligned with the specific circuit process under examination: high-resolution medial temporal lobe imaging and pattern-separation tasks for EC–DG integrity; resting-state fMRI, EEG/ERP, or source-space EEG/MEG connectivity for hippocampal–prefrontal network response; cholinergic-sensitive PET or theta–gamma rhythm measures for MS/VDB–hippocampal function; and actigraphy, sleep timing, and melatonin/cortisol profiles for SCN–PVN-related rhythmicity. In AD models, longitudinal recordings, activity imaging, and circuit-specific manipulations could ascertain whether these biomarkers reflect causal circuit mechanisms. This approach would advance exercise research in early AD from broad cognitive endpoints toward tailored exercise prescriptions, circuit states, and clinically accessible biomarkers.
